# Supplementary material for: The Effectiveness of Compassion Focused Therapy for the Three Flows of Compassion, Self-Criticism, and Shame in Clinical Populations: A Systematic Review
Source: Behav Sci (Basel). 2025 Jul 29;15(8):1031. doi: 10.3390/bs15081031 (PMC12382812; doi:10.3390/bs15081031)
Supplement: Supplementary file 1 [file behavsci-15-01031-s001.zip › behavsci-3703272-supplementary.pdf]

Search Strategy

The following components were included in the search string: search filters – Date.

This search string was constructed by authors (NB) and a librarian. A librarian helped in the design of the search.

Authors (NB) designed and ran the strategy.

No publication type restrictions were applied. We included studies in the following languages: English.

Authors (NB) deduplicated the results.

Authors (NB) conducted the supplementary searches.

Web of Science (all databases)

Search string:
(TI=("Compassion focused" OR "Compassion focussed" OR "CFT" OR "compassionate mind" OR "Compassion based" OR "compassion-based" OR "Compassion Focused Therapy") OR AB=("Compassion focused" OR "Compassion focussed" OR "CFT" OR "compassionate mind" OR "Compassion based" OR "compassion-based" OR "Compassion Focused Therapy") OR AK=("Compassion focused" OR "Compassion focussed" OR "CFT" OR "compassionate mind" OR "Compassion based" OR "compassion-based" OR "Compassion Focused Therapy"))

AND (TI=(Shame OR "internal shame" OR "external shame" OR "self-criticism" OR "self-compassion" OR "compassion") OR AB=(Shame OR "internal shame" OR "external shame" OR "self-criticism" OR "self-compassion" OR "compassion") OR AK=(Shame OR "internal shame" OR "external shame" OR "self-criticism" OR "self-compassion" OR "compassion"))

AND (TI=(intervention OR RCT OR "randomised controlled trial" OR "randomized controlled trial" OR "clinical trial" OR "group therapy" OR "group intervention" OR treatment) OR AB=(intervention OR RCT OR "randomised controlled trial" OR "randomized controlled trial" OR "clinical trial" OR "group therapy" OR "group intervention" OR treatment) OR AK=(intervention OR RCT OR "randomised controlled trial" OR "randomized controlled trial" OR "clinical trial" OR "group therapy" OR "group intervention" OR treatment))

NOT TI=(child OR adolescent) OR AB=(child OR adolescent) OR AK=(child OR adolescent)

Filters (Publication Date): 2000/1/1 - current date (2024/10/05)
Title, Abstract and Authors Keywords

PubMed

Search string: ((((shame[Title/Abstract] OR "internal shame"[Title/Abstract] OR "external shame"[Title/Abstract] OR self-criticism[Title/Abstract] OR “self-compassion”[Title/Abstract] OR “compassion”[Title/Abstract])

OR (shame[Other Term] OR "internal shame"[Other Term] OR "external shame"[Other Term] OR “self-criticism”[Other Term] OR “self-compassion”[Other Term] OR “compassion”[Other Term]))

AND ((intervention[Title/Abstract] OR RCT[Title/Abstract] OR “randomised controlled trial”[Title/Abstract] OR “randomized controlled trial”[Title/Abstract] OR "clinical trial"[Title/Abstract] OR “group therapy”[Title/Abstract] OR “group intervention”[Title/Abstract] OR treatment[Title/Abstract])

OR (intervention[Other Term] OR RCT[Other Term] OR “randomised controlled trial”[Other Term] OR “randomized controlled trial”[Other Term] OR "clinical trial"[Other Term] OR “group therapy”[Other Term] OR “group intervention”[Other Term] OR treatment[Other Term])))

AND (("Compassion focused"[Title/Abstract] OR "Compassion focussed"[Title/Abstract] OR "CFT"[Title/Abstract] OR "compassionate mind"[Title/Abstract] OR "compassion based"[Title/Abstract] OR “compassion-based”[Title/Abstract] OR “Compassion Focused Therapy”[Title/Abstract])

OR ("Compassion focused"[Other Term] OR "Compassion focussed"[Other Term] OR "CFT"[Other Term] OR “compassionate mind”[Other Term] OR "compassion based"[Other Term] OR "compassion-based"[Other Term] OR “Compassion Focused Therapy”[Other Term]))

NOT (child[Title/Abstract] OR adolescent[Title/Abstract] OR child[Other Term] OR adolescent[Other Term]))

Filters: from 2000/1/1 – current date (2024/10/05)
Title, Abstract and Other Terms (Authors Keywords)

APA PsycInfo (EBSCO*host*)

Search string:
(TI (“Compassion focused” OR “Compassion focussed” OR “CFT” OR “compassionate mind” OR “Compassion based” OR “compassion-based” OR “Compassion Focused Therapy”) OR AB (“Compassion focused” OR “Compassion focussed” OR “CFT” OR “compassionate mind” OR “Compassion based” OR “compassion-based” OR “Compassion Focused Therapy”) OR KW (“Compassion focused” OR “Compassion focussed” OR “CFT” OR “compassionate mind” OR “Compassion based” OR “compassion-based” OR “Compassion Focused Therapy”)) AND (TI (Shame OR “internal shame” OR “external shame” OR “self-criticism” OR “self-compassion” OR "compassion") OR AB (Shame OR “internal shame” OR “external shame”

OR “self-criticism” OR “self-compassion” OR "compassion") OR KW (Shame OR “internal shame” OR “external shame” OR “self-criticism” OR “self-compassion” OR "compassion"))
AND (TI (intervention OR RCT OR “randomised controlled trial” OR “randomized controlled trial” OR “clinical trial” OR “group therapy” OR “group intervention” OR treatment) OR AB (intervention OR RCT OR “randomised controlled trial” OR “randomized controlled trial” OR “clinical trial” OR “group therapy” OR “group intervention” OR treatment) OR KW (intervention OR RCT OR “randomised controlled trial” OR “randomized controlled trial” OR “clinical trial” OR “group therapy” OR “group intervention” OR treatment))
NOT TI (child OR adolescent) OR AB (child OR adolescent) OR KW (child OR adolescent)

Filters: from 2000/1/1 - current date (2024/10/05)
Title, Abstract and Keywords
